# CircEpc1 Promotes Ricin Toxin-Induced Inflammation via Activation of NF-κB and MAPK Signaling Pathways by Sponging miR-5114

**DOI:** 10.3389/fphar.2021.767900

**Published:** 2021-10-22

**Authors:** Mingxin Dong, Xiaohao Zhang, Haotian Yu, Yan Wang, Ying Chang, Chengbiao Sun, Jianxu Zhang, Na Zhao, Kaikai Yu, Guangchao Sun, Guiru Zhao, Na Xu, Wensen Liu

**Affiliations:** ^1^ Changchun Veterinary Research Institute, Chinese Academy of Agricultural Sciences, Changchun, China; ^2^ Department of Cardiology, The Second Hospital of Jilin University, Changchun, China; ^3^ Jilin Medical University, Jilin, China; ^4^ Changchun Vocational Institute of Technology, Changchun, China

**Keywords:** ricin toxin, circEpc1, Nod2, NF-κB/MAPK, inflammation

## Abstract

Increasing studies have concentrated on investigating circular RNAs (circRNAs) as pivotal regulators in the progression of numerous diseases and biological processes and abundant evidence shows that circRNAs are participated in the regulation of innate immune responses. Several studies showed that Ricin Toxin (RT) could induce inflammatory injury. There was no research on the particular functions and underlying mechanisms of circRNAs in RT-induced inflammation. In this study, RNA sequencing performed on RT-treated and normal RAW264.7 macrophage cells was used to investigated the differentially expressed circRNAs. Based on the dataset, the expression of circEpc1 (mmu_circ_0,000,842) was identified higher in RT-treated cells. Moreover, gain-and-loss function assays showed that circEpc1 function as a promoter in RT-induced inflammation *in vivo* and *in vitro*. Mechanistically, circEpc1 acted as a miR-5114 sponge to relieve the suppressive effect of miR-5114 on its target NOD2 and thereby activating NF-κB and MAPK signaling pathways. Our results illuminated a link between RT-induced inflammation and the circEpc1 regulatory loop and provided novel insight into the functions of circRNA in innate immune, which may emerge as a potential target in immunotherapy to control the RT-induced inflammatory injury.

## Introduction

Ricin toxin (RT) is a natural plant toxin derived from the seeds of the castor plant (*Ricinus communis*), which is widely cultivated and processed worldwide (Audi et al., 2005). Because of the extreme toxicity of RT, incidents of human and animal poisoning or death due to contact or accidental ingestion of RT have been reported continually (Assiri, 2012). Considering the mechanism of RT cytotoxic, it was identified as type II ribosome-inactivating protein (RIP) (Endo et al., 1987). Moreover, the damage of the 28 S rRNA by RT triggers a specific kinase-activated pathway and induces inflammation (Sowa-Rogozinska et al., 2019). Based on our previous work, RT-treated macrophages release various types of cellular factors, including IL-6, TNF-α, IFN-β, and other cytokines (Liao et al., 2016; Dong et al., 2020). When these cellular events occur, an intracellular signaling cascade is activated through various pattern-recognition receptors (PRRs) spoke on macrophages. One of the most critical PRRs involved in inflammation is nucleotide-binding oligomerization domain 2 (NOD2) which recognizes bacterial cell walls (G. Chen et al., 2009; Gresnigt et al., 2018). Activation of NOD2 results in activation of multiple signaling pathways, such as NF-κB and MAPK pathways, and ultimately leads to types of inflammation responses.

CircRNAs, a novel class of non-coding RNAs, as a type of regulatory RNAs have attracted extensive research interest. Structurally, circRNAs are characterized by closed continuous loop structures with neither 5′- 3′ polarity nor a polyadenylated tail (Jeck and Sharpless, 2014). The majorities of circRNAs are abundant, highly conserved across species, and tissue or developmental-stage specific (Salzman et al., 2013; Rybak-Wolf et al., 2015). Compared with their linear counterparts, circRNAs exhibit higher stability due to the loop structure being resistant to RNase R *in vivo* (Li et al., 2015).

Currently, with the rapid development of high-throughput sequencing techniques and bioinformatics, RNomics has gradually become a focus of attention. Thus, accumulated knowledge of characteristics and functions of circRNAs is beginning to be understood. Studies have shown that circRNAs play essential roles in human diseases, including atherosclerotic vascular disease (Holdt et al., 2016), neurological disorders (Lukiw, 2013), cardiovascular disease (Lei et al., 2018), and cancer (Guarnerio et al., 2016a; 2016b). Emerging evidence has shown that circRNAs have been identified as competing endogenous RNAs (ceRNAs) that function as miRNA sponges via complementary base paring (Hansen et al., 2013). For instance, the circRNA ciRS-7 inhibits miRNAs in murine tissue by sponging miR-7 (Memczak et al., 2013). Additionally, besides the ceRNA mechanism, circRNAs can also interact with RNA-binding proteins (RBP) to regulate target gene expressions and some of them can encode functional proteins (Du et al., 2016; Legnini et al., 2017; Yang et al., 2018). These studies strongly confirm that circRNAs play a fundamental role in a variety of cellular processes. Our previous study have confirmed that RT can cause inflammation in RAW264.7 macrophage cells (Xu et al., 2013). Researches have showed that circRNAs plays an important role in the occurrence and progression of many diseases. However, the biological effects and underlying mechanisms of circRNAs in RT-induced inflammation have not been explored comprehensively.

In this study, we investigate the expression profiling of circRNAs in RT-treated and normal RAW264.7 macrophage cells by RNA-Seq. We found a significant upregulated circRNA, mmu_circ_0,000,842, designated as circEpc1, is initially identified which may promote inflammation. Mechanistically, circEpc1 functions as miRNA sponge to regulate the expression of NOD2 by competitive binding to miR-5114, leading to the activation of NF-κB and MAPK pathways. Collectively, our results revealed that the circEpc1/miR-5114/NOD2 axis has a considerable role in RT-induced inflammation, identifying circEpc1 as a potential biomarker and therapeutic target for RT-induced inflammatory injury.

## Materials and Methods

### Cell Line and Culture

Mouse mononuclear macrophage cell line RAW264.7 was purchased from the American Type Culture Collection (ATCC, VA, USA). The cells were cultured at 37 C in a humidified atmosphere with 5% CO_2_ and maintained in RPMI-1640 medium (Gibco, CA, United States) supplemented with 10% fetal bovine serum (FBS) (HyClone, UT, United States), 100 U/ml penicillin and 100 U/ml streptomycin (Invitrogen, CA, United States).

### circRNA Library Construction and Sequencing

RAW264.7 cells were prepared as described previously (Dong et al., 2020). RT (20 ng/ml) was used to treat cells for 8 h. Total RNA was extracted using TRIzol reagent (Takara, Tokyo, Japan) following the manufacturer’s instructions. The total RNA quantity and purity were analysis of Bioanalyzer 2,100 and RNA 6000 Nano LabChip Kit (Agilent, CA, United States) with RIN number >7.0. Approximately 10 ug of total RNA representing a specific adipose type was used to deplete ribosomal RNA according to the manuscript of the Epicentre Ribo-Zero Gold Kit (Illumina, San Diego, United States). Following purification, the poly (A)- or poly(A)+ RNA fractions is fragmented into small pieces using divalent cations under elevated temperature. Then the cleaved RNA fragments were reverse-transcribed to create the final cDNA library in accordance with the protocol for the RNA-Seq sample preparation kit (Illumina, San Diego, USA), the average insert size for the paired-end libraries was 300 bp (±50 bp). And then we performed the paired-end sequencing on an Illumina Hiseq4000 at the (LC Sciences, USA) following the vendor’s recommended protocol. CircRNA expressions from different samples or groups were calculated by scripts in house. Only the comparisons with *p* < 0.05 were regarded as showing differential expression.

### RNA Extraction, Reverse Transcription, and qRT-Polymerase Chain Reaction Analysis

Total RNA was extracted from cultured cells and lung tissue using TRIzol reagent (Takara, Tokyo, Japan) according to the manufacturer’s instructions. gDNA was extracted using Genomic DNA Isolation Kit (Sangon Biotech, Shanghai, China). Reverse transcription was performed using Oligo (dT) primer for mRNA into cDNA with M-MLV Reverse Transcriptase, RNase H- (Takara, Tokyo, Japan). A Transcriptor First Strand cDNA Synthesis Kit (Roche, Basel, Switzerland) was used to verify the existence of circRNAs. The qRT-PCR was conducted using Applied Biosystems QuantStudio three Real-Time PCR Systems (Thermo Fisher Scientific, MA, United States) with SYBR Green PCR Master Mix (Takara, Tokyo, Japan). The expression levels of miRNA were determined using an All-in-One miRNA qPCR Kit (GeneCopoeia, MD, United States). β-actin was measured as an endogenous control for mRNA and circRNA, and U6 was used as a control for miRNA. The relative fold-change in expression with respect to a control sample was calculated by the 2^−ΔΔCt^ method. Relative primers are shown in Supplementary Table S1.

### RNase R Treatment

2 μg of total RNA was incubated with or without 5 U/μg RNase R at 37 °C for 30 min in the buffer provided with the kit (Epicentre Technologies, Wisconsin, United States), and subsequently purified by RNeasy MinElute Cleaning Kit (Qiagen), then analyzed via RT-PCR.

### Nucleocytoplasmic Separation and RNA Isolation

PARIS Kit (Invitrogen, CA, United States) was used to separately isolate nuclear and cytoplasmic RNA from cultured cells, according to the manufacturer’s protocol.

### Actinomycin D Assay

RAW264.7 cells were exposed to 2 μg/ml actinomycin D (Sigma Aldrich, St. Louis, MO, United States) at indicated time point. Then the cells were harvested, and total RNA was extracted. The stability of circEpc1 and Epc1 mRNA was analyzed using qRT-PCR.

### Lentivirus Vector Constructs and Cell Transfection

For *in vitro* studies, the lentivirus vectors were constructed using the pHBLV-CMV-Circ-MCS-EF1-ZsGreen-T2A-PURO vector purchased from Hanbio Biotechnology (Shanghai, China), and further confirmation was obtained by Sanger Sequencing. The circEpc1 overexpressing lentivirus vector, two shRNAs (sh-circEpc1#1 and sh-circEpc1#2) targeting circEpc1, and miR-5114 mimics were also designed and synthesized by HanBio. Cell transfection was performed as we described previously (Dong et al., 2020). Then, 5 μg/ml of Puromycin (MedChemExpress, NJ, United States) was used to select the stably infected cells. The transfection efficiency was determined by qRT-PCR.

### Dual-Luciferase Reporter Assay

Luciferase assays were performed using the DLR Assay System (Promega, WI, USA) according to the manufacturer’s instructions. Briefly, the HEK293T cells were co-transfected by Lipofectamine 3,000 (Invitrogen, CA, United States) with miR-5114 mimics or negative control (NC) mimics (Sequence: 5′-AGA​ACG​UCG​AAG​GCA​GAG​GUC​A-3′) and luciferase reporter constructs containing the wild-type or mutant 3′-UTR of circEpc1. The mimics and luciferase reporter constructs were purchased from Hanbio (Shanghai, China). The cells were lysed 24 h after transfection, then Renilla luciferase (RLuc) and Firefly luciferase (Fluc) activities were measured on a Synergy 2 luminometer (BioTek, USA). Rluc signals were normalized to the intraplasmid Fluc transfection control.

### Fluorescence *in situ* Hybridization (FISH)

For FISH, the RT-treated RAW264.7 cells were incubated using specific probes of circEpc1 and miR-5114 according to user manual of RNA FISH Kit (GenePharma, Shanghai, China). The cells and fluorescence-labeled probes were hybridized in a hybridization buffer and hybridized overnight at 37°C. The next day, after stringent washing with SSC buffer, the nucleus were counterstained with DAPI. Images were acquired using a LSM-780 confocal laser scanning microscope (Carl Zeiss, Germany) and digitized with a software program Zen Light Edition.

### Animal Experiments

Male BALB/c mice aged 6–8 weeks, weighting 20–25 g, were purchased from the Liaoning Changsheng biotechnology Co. Ltd. (Benxi, China). To explore the regulatory function of circEpc1 *in vivo*, the adeno-associated virus (AAV) circEpc1 overexpressing, shRNA targeting circEpc1, and (NC) were constructed and packaged by HanBio. The mice were randomly divided into three groups (NC, circEpc1-overexpression, and sh-circEpc1) with eight mice in each group. One week after the mice have acclimated to the environment, a total of 50 µl solution containing above virus or RT was slowly injected into trachea by means of liquid aerosol lung delivery. All mice were sacrificed 3 weeks after the injection, and the lungs were cut out for histopathological analysis.

### *Ex vivo* Fluorescence Imaging

In order to detect the transfection effect of AAVs *in vivo*, we carried out *Ex vivo* tissue fluorescence imaging experiment. Then, under anesthesia, 3 weeks after AAVs transfection, the imaging system was used for fluorescence imaging of lung tissue in mice. Maestro software was used to remove the mouse background fluorescence.

### Histopathological Analysis

Histopathological analysis was performed according to the manufacturer’s protocol. After injection, the mouse lungs were cut out and immersed in 4% paraformaldehyde overnight, embedded in paraffin and then sections Discussion μm thick were cut. The sections were dehydrated in xylene and ethanol successively. Hematoxylin-eosin (H&E) staining was performed to evaluate the morphological variation in lung and inflammatory cell infiltration. Sections were microscopically examined using Nikon (Eclipse 80i; Tokyo, Japan), and the images were acquired on the imaging system (digital sight DS-FI2, Nikon, Japan).

### Western Blot Analysis

Protein of RT-treated RAW264.7 cells and lung tissue were homogenized in RIPA lysis and extraction buffer (Thermo Fisher Scientific, MA, United States) supplemented with PMSF. A BCA Protein Assay Kit (Beyotime Biotechnology, Shanghai, China) was used to quantify the concentration of protein samples in the cell lysates. Then, the proteins were separated by 10% SDS-PAGE and electroblotted onto polyvinylidene difluoride (PVDF) membranes (Millipore, Billerica, MA, United States). Following blocking the PVDF membranes with 5% nonfat milk (BD Biosciences, Franklin Lakes, NJ, United States) in 0.1% Tween-20 TBST buffer at room temperature for 1 h, the membranes incubated with antibodies included NOD2 (1:2000, proteintech, 66,710), RIP2 (1:1,000, Abcam, ab8428), p65 (1:2000, proteintech, 66,535), IκB-α (1:4,000, Abcam, ab32518), P-IκB-α (1:1,000, CST, 28,595), p38 (1:2,500, BD Biosciences, 612,280), p-p38 (1:5,000, BD Biosciences, 612,168), ERK (1:4,000, BD Biosciences, 610,030), p-ERK (1:1,000, BD Biosciences, 612,358), JNK (1:250, BD Biosciences, 612,540), p-JNK (1:250, BD Biosciences, 610,627) overnight at 4°C. β-actin (1:10,000, proteintech, 66,009) was used as the internal protein loading control. After being washed 5 times in TBST for 50°min, membranes were incubated with HRP-labelled secondary antibody at room temperature for 1 h. Finally, immunodetection was performed using an enhanced chemiluminescence (ECL) detection system with Chemiluminescence HRP Substrate (GE Healthcare, Buckinghamshire, United Kingdom).

### Cytokine Assays

After RT treated, mice lungs were irrigated three times with 1.0 ml PBS. The collected bronchoalveolar lavage fluid (BALF) was immediately centrifuged to separate the cells and supernatant. Measurement of cytokine in the supernatants of BALF and RT-treated RAW264.7 cells was carried out using Mouse TNF-α ELISA kit (Dakewe, Shenzhen, China) and Mouse IL-6 ELISA kit (Biolegend, San Diego, CA, United States) according to the manufacturer’s instructions.

### Statistics

We performed our experiments in triplicate, and the results are presented as mean ± standard deviation of the mean. Statistical analyses were performed using GraphPad Prism 8 (GraphPad Software, Inc. La Jolla, CA), and consisted of analysis of variance followed by Student’s t-test when comparing two experimental groups. p < 0.05 was considered statistically significant, *p* < 0.01 and *p* < 0.001 were considered indicative of highly significant difference.

## Results

### Differential Expression of circRNAs in RT-Treated RAW264.7 Macrophage Cells

**FIGURE 1:**
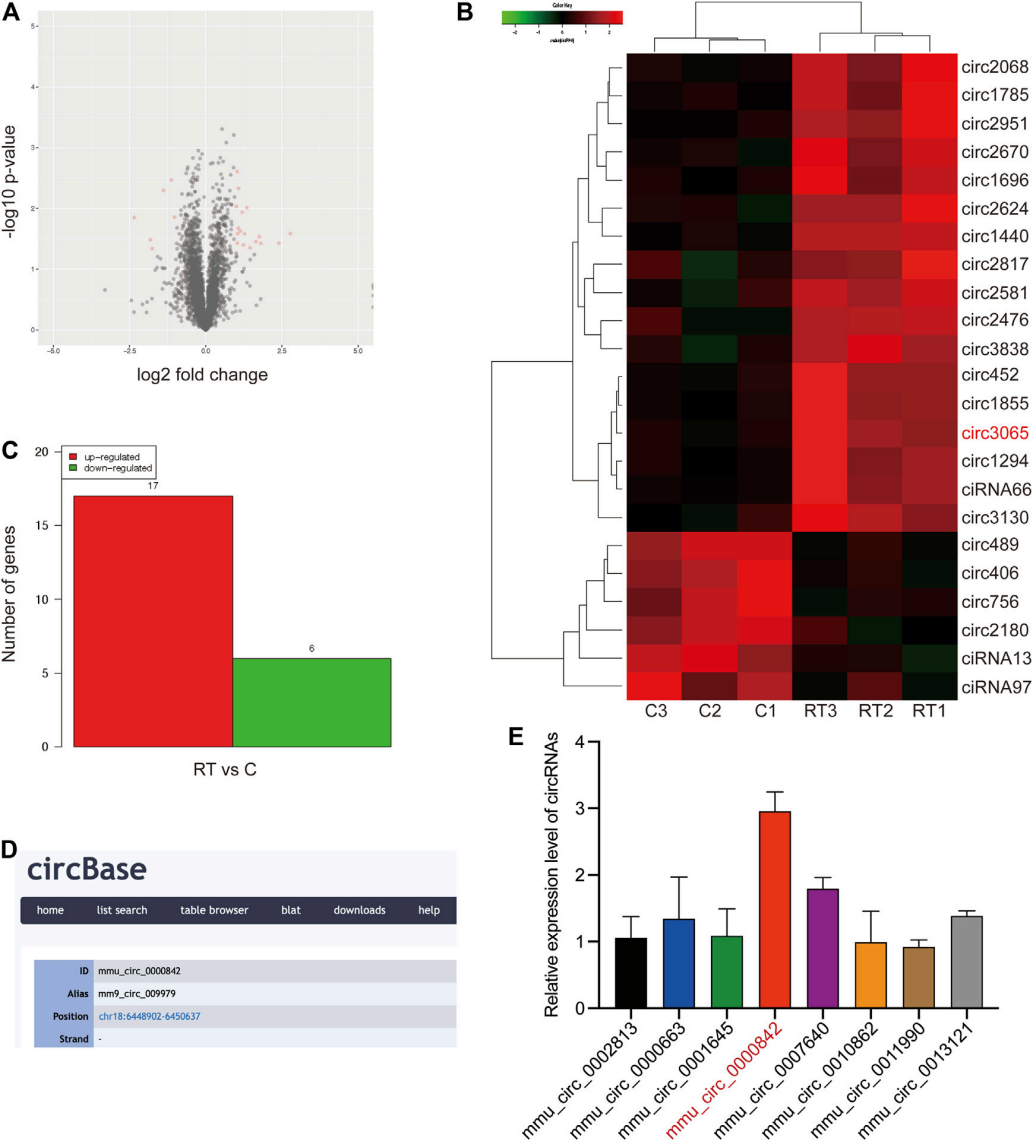
Identification of circRNAs expression profile. **(A)** Volcano plots were constructed using fold change values and p-values. The red dot in the plot represents the significantly different expression (fold change≥ 2, p < 0.05) of circRNAs showing statistical significance. **(B, C)** Hierarchical clustering analysis of circRNAs that are significantly differentially expressed in control and RT-treated RAW264.7 cells, including 17 upregulated and six downregulated circRNAs; each group contained three biological repeats (fold change≥ 2, p < 0.05). The expression levels are presented in different colors indicating expression levels above and below the median expression level across all samples. **(D)** ID number and alias of mmu_circ_0,000,842 from the circbase database. **(E)** Expression levels of top8 significantly differentially expressed circRNAs were determined by qRT-PCR.

To identify circRNAs that are crucial to RT-induced inflammation, RNA-seq was performed for RT-treated RAW264.7 macrophage cells and matched normal RAW264.7 macrophage cells. As shown in Figure 1A, a total of 4,273 dysregulated circRNAs were identified in RT-treated RAW264.7 cells, of which 1,634 circRNAs were upregulated and 2,639 circRNAs were downregulated. In addition, we found that 17 circRNAs were significantly upregulated, and six circRNAs were significantly downregulated in RT-treated RAW264.7 cells (filtered by fold change ≥2 and *p* < 0.05) (Figures 1B,C).

Among these circRNAs, we focused on the top eight significantly differentially expressed circRNAs to verify their abundance in RT-treated cells based on their fold change. Interestingly, a circRNA (circRNA ID in circbase: mmu_circ_0,000,842, http://www.circbase.org/, termed circEpc1 in the remainder of the article), which was derived from the Epc1 gene locus and most highly upregulated circRNA attracted our attention (Figure 1D). To further investigate the expression level of circEpc1 according to the RNA-seq data, we detected higher circEpc1 expression in RT-treated RAW264.7 cells via qRT-PCR, which was consistent with the RNA-seq data (Figure 1E). Taken together, these results suggested that circEpc1 as an upregulated circRNA participate in RT-induced RAW264.7 macrophage inflammation.

### Identification of the Circular Structure of circEpc1

**FIGURE 2:**
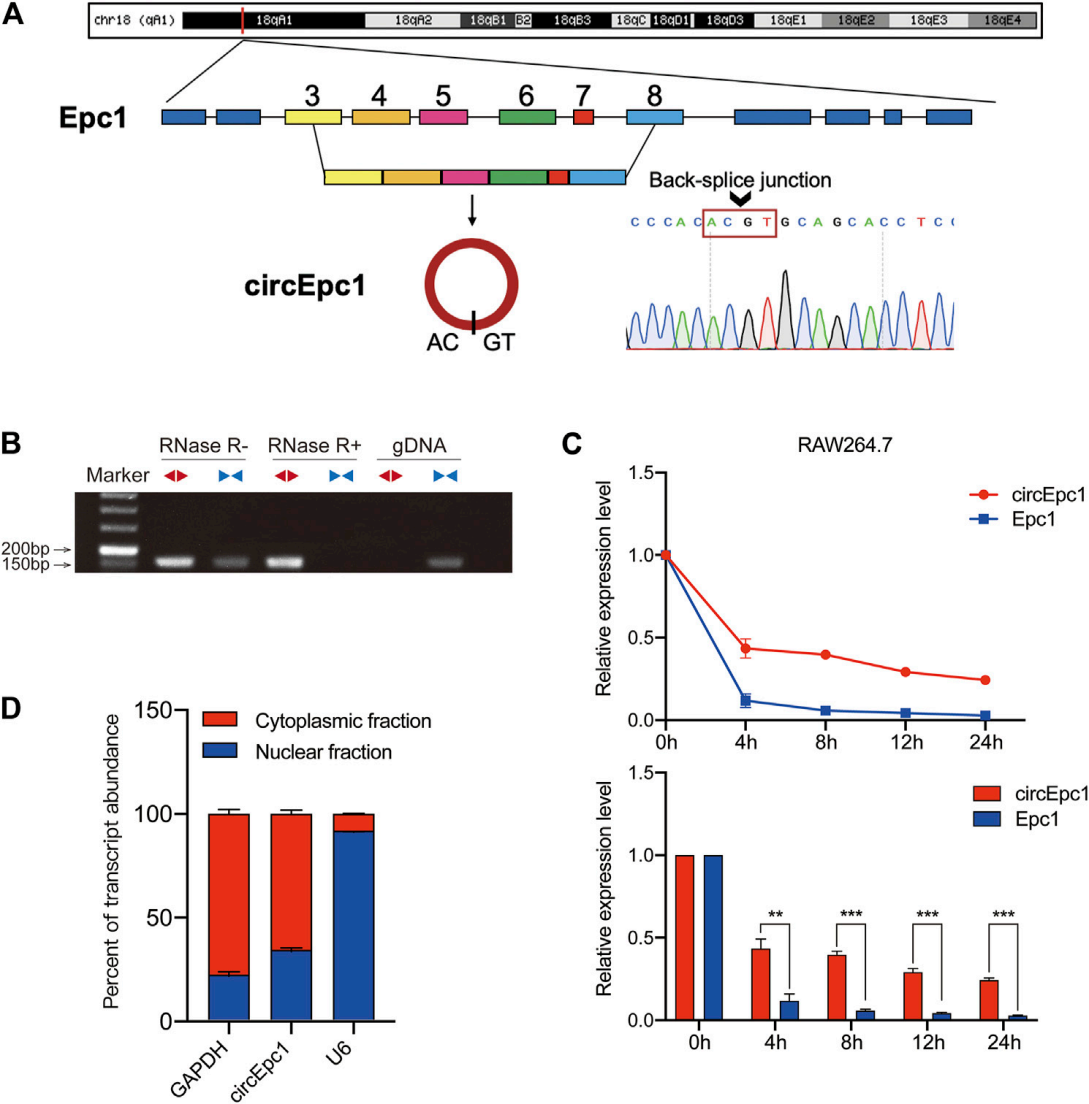
Characterization of circEpc1 in RT-treated RAW264.7 cells. **(A)** Genomic loci of circEpc1 gene. circEpc1 is produced at the Epc1 gene (ID: 13,831) locus containing exons 3–8. The back-splice junction of circEpc1 was identified by Sanger sequencing. **(B)** RT-PCR analysis for circEpc1 and its linear isoform Epc1 in cDNA and gDNA in the presence or absence of RNase R supplementation from RAW264.7 cells using divergent and convergent primers. **(C)** qRT-PCR for the abundance of circEpc1 and Epc1 in RAW264.7 cells treated with Actinomycin D at the indicated time point. **(D)** Levels of circEpc1 in the nuclear and cytoplasmic fractions of RAW264.7 cells. Data represent the mean ± SD from three representative experiments (*p < 0.05, **p < 0.01, ***p < 0.001).

CircEpc1 arises from the Epc1 gene, which is located at chromosome 18 and consists of the head-to-tail splicing of exon three and exon 8 (6,448,902–6,455,334). Sanger sequencing was performed to validate its back-splicing using the RT-PCR product of circEpc1. The sequence is consistent with circbase database annotation (http://www.circbase.org/) (Figure 2A). To further confirm the circular form of circEpc1, we designed divergent and convergent primers to amplify the circular and linear forms of Epc1, respectively. RT-PCR was performed to detected the expression level of circular and linear forms of Epc1 with or without RNase R supplementation in the reverse-transcribed RNA (cDNA) and genomic DNA (gDNA) of RAW264.7 cells. Results showed that divergent primers could amplify products from cDNA but not from gDNA (Figure 2B). Moreover, analysis for stability of circEpc1 and linear Epc1 in RAW264.7 cells treated with Actinomycin D, an inhibitor of transcription, showed that circEpc1 was more stable than linear Epc1 (Figure 2C). In addition, the nuclear-cytoplasmic fractionation results revealed that circEpc1 was primarily located in the cytoplasm rather than nuclear (Figure 2D). These results further confirm the characteristics of circEpc1 as a circRNA and imply that its function may be benefited from the biological stability.

### CircEpc1 Acts as a Facilitator in RT-Induced Inflammation of RAW264.7 Cells

**FIGURE 3:**
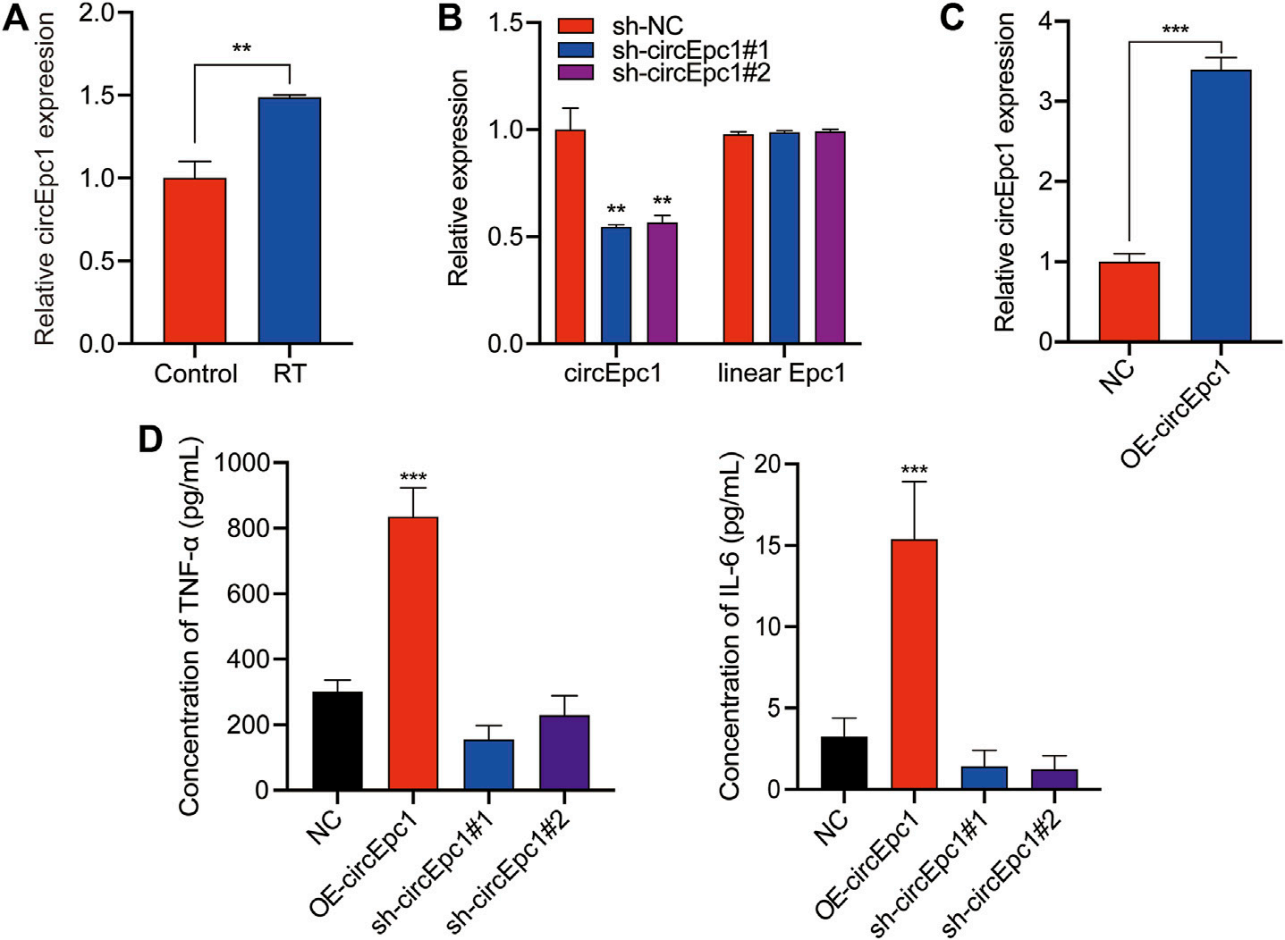
Characterization of circEpc1 in RT-treated RAW264.7 cells. **(A)** Expression level of circEpc1 in control and RT-treated RAW264.7 cells. **(B)** Two shRNAs were designed to silence circEpc1 by targeting the spliced junction of circEpc1, it revealed that the shRNAs could significantly downregulate the expression level of circEpc1 but had no effect on that of linear Epc1. (C) Expression level of circEpc1 in RAW264.7 cells after transduction with over-expressing circEpc1 lentivirus. **(D)** ELISA assays were performed to determine the role of circEpc1 in RT-induced inflammation. Data represent the mean ± SD from three representative experiments (*p < 0.05, **p < 0.01, ***p < 0.001).

Next, we examined the expression of circEpc1 in RT-treated and normal RAW264.7 cells via qRT-PCR. The expression of circEpc1 was significantly increased in RT-treated cells (Figure 3A). Meanwhile, based on our previous work, ELISA assays showed that the secretion of TNF-a and IL-6 in the supernatant of RT-treated cells was significantly higher than that of the control group (Dong et al., 2020). Collectively, these results suggested that circEpc1 may be involved in RT-induced inflammation.

To investigate the role of circEpc1 in RT-induced inflammation, two small hairpin RNAs (sh-circEpc1#1 and sh-circEpc1#2) were constructed to target the unique back-splicing junction of circEpc1. The shRNAs significantly decreased circEpc1 expression without decreasing the linear Epc1 mRNA level. In addition, RAW264.7 cells were also transfected with the circEpc1 lentivirus vector to overexpress circEpc1. The efficiency and specificity of circEpc1 knockdown and overexpression in RAW264.7 cells verified by qRT-PCR (Figures 3B,C). However, after knockdown of circEpc1, the secretion of TNF-α and IL-6 was significantly decreased. On the contrary, the overexpression of circEpc1 showed the opposite result. (Figure 3D). Taken together, these data suggest that circEpc1 played as a facilitator in RT-induced inflammation.

### MiR-5114 Is Sponged by circEpc1 and Plays a Negative Role in RT-Induced Inflammation

**FIGURE 4:**
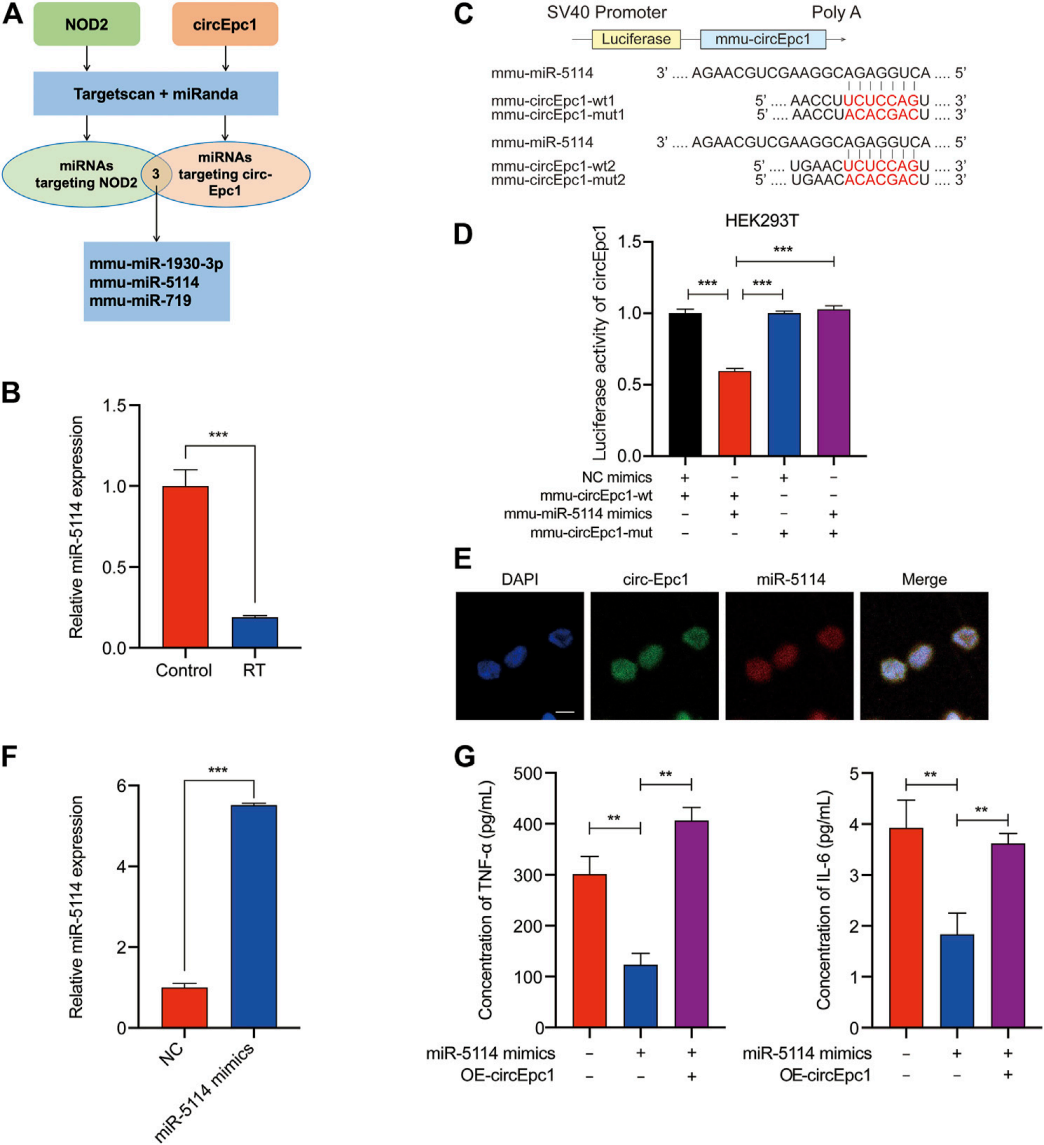
CircEpc1 serves as a miRNA sponge of miR-5114. (**A)** The schematic flowchart shows the pipelines of miRNAs which could bond to circEpc1 and NOD2 3′-UTR via online bioinformatic network. (**B)** Expression level of miR-5114 in control and RT-treated RAW264.7 cells. (**C, D)** Dual-luciferase reporter activity of circEpc1 in HEK-293T cells co-transfected with miR-5114 mimics or mimics NC. (**E)** MiR-5114 co-localized with circEpc1 in the cytoplasm by FISH analysis, scale bar = 100 μm **(F)** Expression level of miR-5114 in RAW264.7 cells after transduction with miR-5114 mimics lentivirus. **(G)** TNF-a and IL-6 secretion in RT-treated RAW264.7 cells transfected with miR-5114 mimics alone or co-transfected with circEpc1. Data represent the mean ± SD from three representative experiments (*p < 0.05, **p < 0.01, ***p < 0.001).

Extensive studies have reported that circRNAs mostly function as miRNAs sponge to regulate downstream genes (Han et al., 2017; Yu et al., 2018). Therefore, taking the sequence of circEpc1 as a bait, we constructed a circEpc1-miRNAs-NOD2 network and performed a cross-analysis using two miRNA target prediction online databases (TargetScan and miRanda). This network included three candidate miRNAs (miR-1930–3p, miR-5114, and miR-719), containing common binding sites for the circEpc1 and NOD2 (Figure 4A). Based on our previous work, RNA-Seq technology was used to perform an analysis of the miRNA profiles of RT-treated RAW264.7 macrophage cells. Interestingly, we found that the expression of miR-5114 was significantly downregulated and the same results were obtained by qRT-PCR assays in RT-treated RAW264.7 cells (Figure 4B), suggesting that there may be a regulatory relationship between circEpc1 and miR-5114.

To confirm miR-5114 could be regulated by circEpc1, a dual-luciferase reporter assay was used to determine the direct binding between circEpc1 and miR-5114 based on their complementary sequences. We constructed luciferase reporters containing wild type and mutated putative binding sites of circEpc1 transcripts (Figure 4C). Then, we co-transfected a miR-5114 mimic with the reporter gene into HEK293T cells. Results of luciferase reporter assays showed that the luciferase activities of circEpc1 wild type reporter were significantly reduced when transfected with miR-5114 mimics compared with control reporter or mutated luciferase reporter (Figure 4D). Thus, the direct interaction between circEpc1 and miR-5114 was confirmed. Furthermore, FISH analysis was performed in RT-treated RAW264.7 cells, and we found that miR-5114 was co-localized with circEpc1 in the cytoplasm, which further verification the interaction between circEpc1 and miR-5114 (Figure 4E).

To study the function of miR-5114, miR-5114 mimics was separately transfected into RAW264.7 cells and its effect on the expression of miR-5114 was detected (Figure 4F). Moreover, we found that, miR-5114 mimics significantly suppressed the TNF-a and IL-6 secretion, and this effect could be reversed after transfection with circEpc1 overexpressing lentivirus vector (Figure 4G). In conclusion, these results revealed that miR-5114 played a negative role in RT-induced inflammation and circEpc1 serves as a sponge of miR-5114.

### CircEpc1 Facilitates RT-Induced Inflammation by Upregulating the Expression of NOD2 and Activating the NF-κB and MAPK Signaling Pathways

With the aim to detect the detailed mechanism of circEpc1 in RT-induced inflammation, we focused on a gene involved in inflammation responses to get some key clues. Increasing evidences demonstrate that NOD2, an intracellular PRR, plays an important role in innate immune regulation. Once activated, NOD2 oligomerizes and interacts with the serine/threonine kinase receptor-interacting protein 2 (RIP2) though the CARD domains, resulting in the activation of NF-κB and MAPK signaling pathways (Hasegawa et al., 2008; Tao et al., 2009; Fridh and Rittinger, 2012). Here, we investigated whether there is interaction between circEpc1, miR-5114 and NOD2.

**FIGURE 5:**
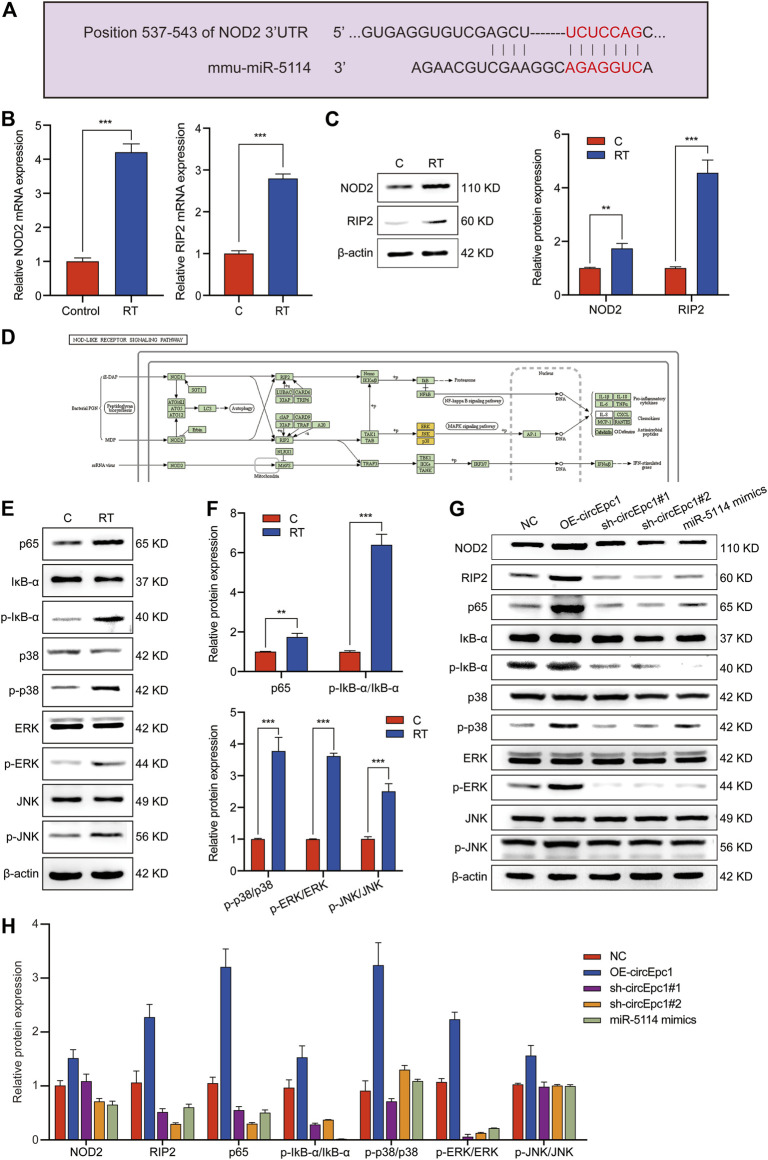
CircEpc1 facilitates RT-induced inflammation by relieving repression of miR-5114 for NOD2 expression in RAW264.7 cells. (**A)** The binding sites of miR-5114 and NOD2 were predicted by TargetScan database. (**B)** Expression level of NOD2 and RIP2 in control and RT-treated RAW264.7 cells. (**C)** The protein expression level of NOD2 and RIP2 in control and RT-treated RAW264.7 cells. **(D)** NOD-Like Receptor signaling pathway. (**E**, **F)** Western blot assays were used to evaluate the activation of MAPK and NF-κB signaling pathways in RAW264.7 cells induced by RT. (**G, H)** Western blot assays were used to evaluate the effects of circEpc1 and miR-5114 on NOD2, RIP2, MAPK and NF-κB signaling pathways in RT-induced inflammation. The intensity of bands were scanned and measured by ImageJ software, and were summarized as bar graphs. Data represent the mean ± SD from three representative experiments (*p < 0.05, **p < 0.01, ***p < 0.001).

Online bioinformatics predictions (TargetScan) indicated that miR-5114 possesses binding sites that are potentially complementary to NOD2 (Figure 5A). Intriguingly, NOD2 and RIP2 were both significantly increased in RT-treated RAW264.7 cells at mRNA and protein levels according to the results of qRT-PCR and Western blot assays (Figures 5B,C), suggesting that NOD2 expression was positively correlated with the circEpc1 expression level in RT-induced inflammation. According to the NOD-Like Receptor signaling pathway shown in Figure 5D, NOD2 mediates the expression of inflammatory cytokines by activating downstream NF-κB and MAPK signaling pathways. Thus, we confirmed this signal transduction mechanism in RT-treated RAW264.7 cells (Figures 5E,F). Furthermore, the regulatory function of circEpc1 was also confirmed in lentivirus-constructed cell lines (Figures 5G,H). Sum up the above results, CircEpc1 facilitates RT-induced inflammation by upregulating the expression of NOD2 and activating the NF-κB and MAPK signaling pathways.

### CircEpc1 Promotes RT-Induced Inflammation *in vivo*

**FIGURE 6:**
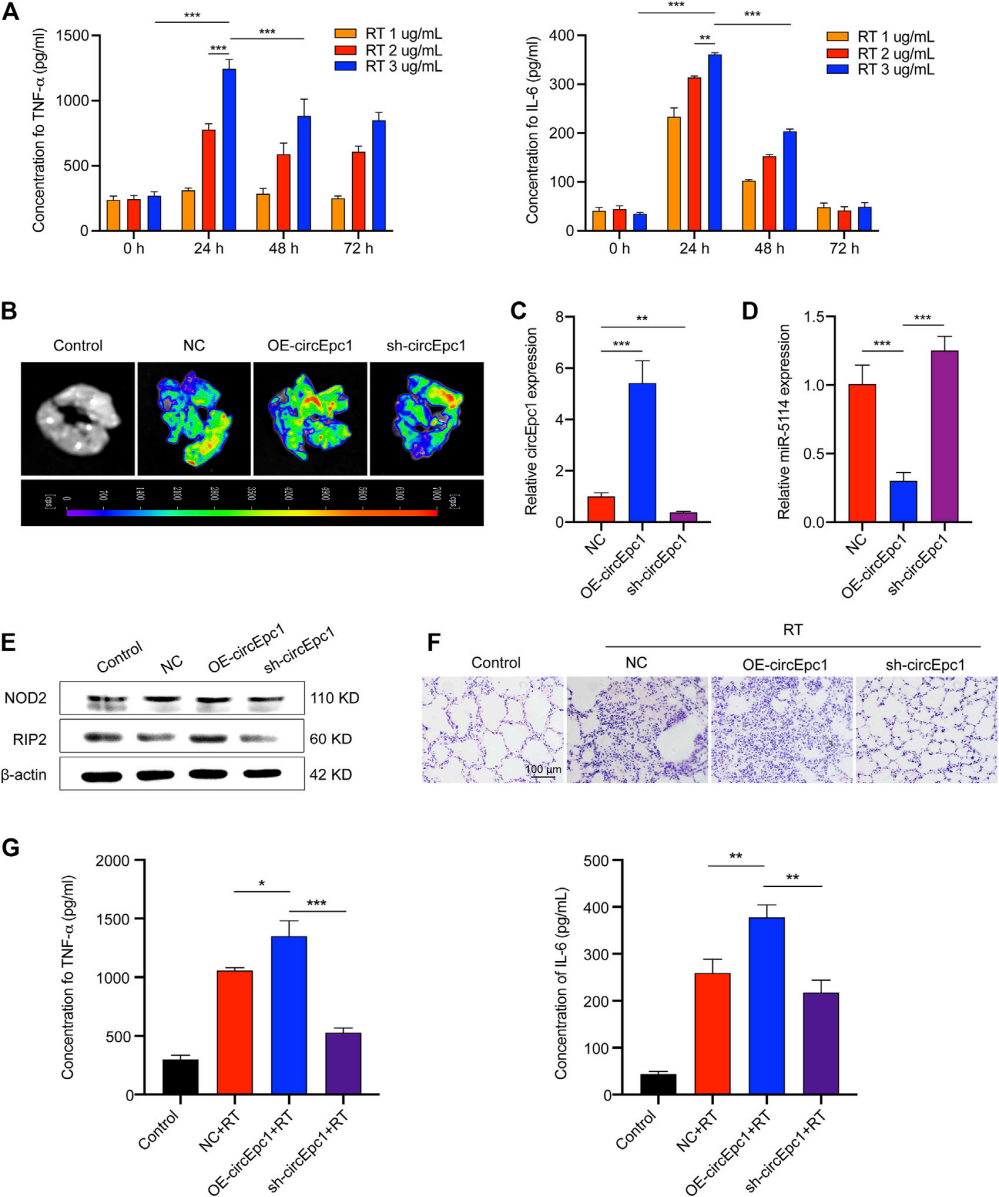
CircEpc1 promotes RT-induced inflammation *in vivo*. (**A)** Effects of RT concentration and exposure time on TNF-α and IL-6 secretion in mice. (**B)** *Ex vivo* fluorescence imaging of lung tissue in control and AAVs transfected mice groups. (**C, D)** qRT-PCR assays were used to determine the expression of circEpc1 and miR-5114 in AAVs transfected mice. (**E)** Western Blot assays were used to determine the protein expression level of NOD2 and RIP2 in AAVs transfected mice. (**F)** The image (H&E staining) of mouse lung morphological changes and inflammatory cell infiltration in control and RT exposed groups of AAVs transfection model mice (scale bar: 100 μm). (**G)** CircEpc1 promotes the secretion of TNF and IL-6 in mice exposed to RT. Data represent the mean ± SD from three representative experiments ( *p < 0.05, **p < 0.01, ***p < 0.001).

To evaluate the contribution of circEpc1 to RT-induced inflammation *in vivo*, the following animal experiment was conducted. Mice were challenged with RT by means of liquid aerosol lung delivery, and the effects of challenge dose and time on the secretion of TNF-α and IL-6 in BALF of mice were evaluated via ELISA (Figure 6A). Based on the results, treatment with RT (2 µg/ml) for 24 h was took as the condition of the follow-up animal experiment. To further investigate the effects of circEpc1 *in vivo*, gain-and-loss function assays were performed through AAV transfection system. Mice were randomly divided into the following three groups: NC, OE-circEpc1, and sh-circEpc1. The transfection effect was detected by *ex vivo* fluorescence imaging system and qRT-PCR after transfection 3 weeks (Figures 6B,C). Meanwhile, overexpression of circEpc1 significantly suppressed the expression of miR-5114 (Figure 6D). In the case of exposed to RT (2 µg/ml), the protein level of NOD2 and RIP2 were significantly upregulated in OE-circEpc1 group, but showed the opposite trend in sh-circEpc1 group (Figure 6E). Furthermore, to investigate the potential pro-inflammatory function of circEpc1, H&E staining was used to detect inflammatory injury in lung tissue (Figure 6F). Following RT exposure, the alveolar wall was thickened, there was extensive neutrophil infiltration, and a large number of inflammatory cell foci appeared around the blood vessels. It was further showed that circEpc1 could significantly promote the secretion of TNF-α and IL-6 (Figure 6G). These results suggested that circEpc1 functions as a promoter in RT-induced inflammation in mice.

## Discussion

Currently, due to the supreme cytotoxicity of RT, it is still a great challenge to deal with the severe inflammatory injury caused by inhaled RT. However, although significant improvements have been made in diagnosis and treatment strategies, there is no effective and approved treatment for RT inhalation poisoning, so new and effective treatments for this injury are needed. Growing evidence indicates that circRNAs, as novel noncoding RNAs, play important roles in multiple inflammatory responses and may be involved in the pleiotropic modulation of cellular functions (X. Li et al., 2020; Li et al., 2019). Thus, it is essential to analyze the differentially expressed circRNAs in RT-treated RAW264.7 macrophage cells and elucidate its basic mechanism in RT-induced inflammation.

**FIGURE 7:**
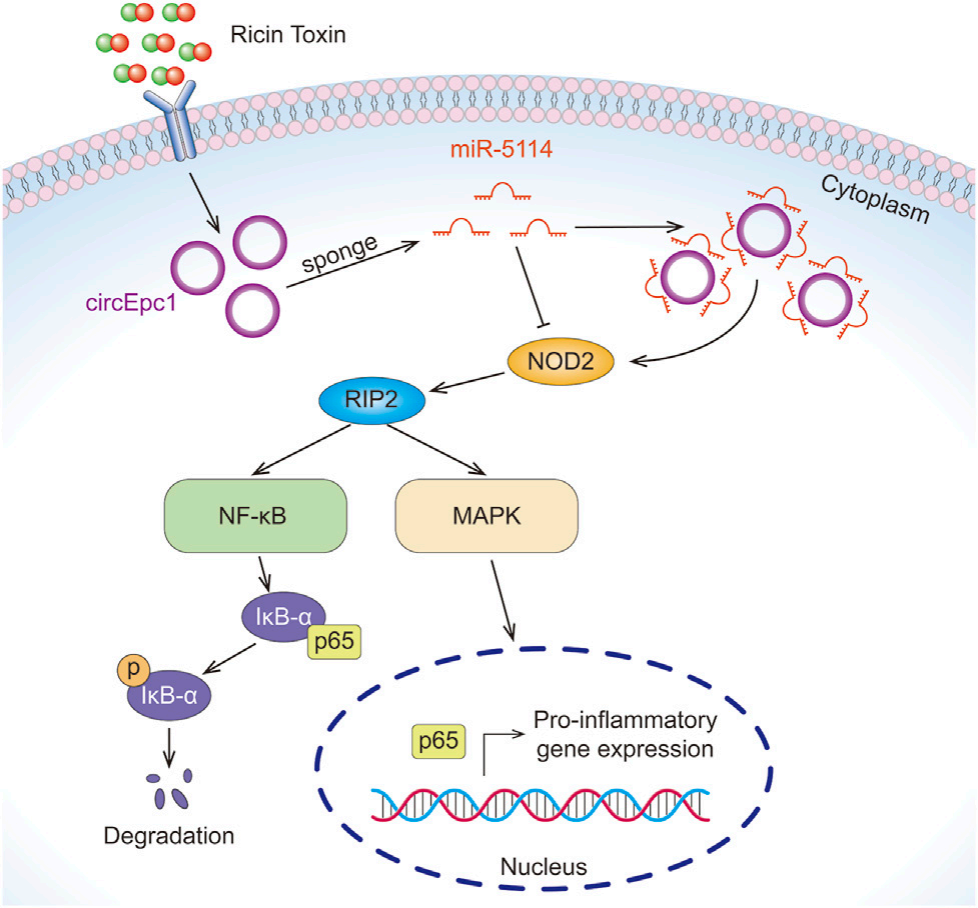
The mechanism of circEpc1 promotes RT-induced inflammation through the circEpc1/miR-5114/NOD2 axis.

In the present study, we profiled circRNA expression in RT-treated and normal RAW264.7 macrophage cells by RNA-Seq, resulting in the identification of 23 significantly differentially expressed circRNAs, including 17 upregulated and six downregulated circRNAs. We identified circEpc1 as a significantly upregulated circRNA in RT-treated RAW264.7 cells and RT-injured mice. Gain-and-loss function assays suggested that the circEpc1 promotes RT-induced inflammation through circEpc1/miR-5114/NOD2 axis (Figure 7).

Our circRNA high-throughput sequencing and qRT-PCR results showed that circEpc1 is significantly overexpressed in RT-treated RAW264.7 cells compared with the normal RAW264.7 cells, and it was the first time to scan circRNAs which play a promoting role in RT-induced inflammation. To elucidate the detailed mechanism underlying circEpc1 function as a facilitator in RT-induced inflammation, we constructed cell lines with circEpc1 overexpression and knockdown using lentiviral vectors. CircEpc1 overexpression significantly promotes the secretion of TNF-α and IL-6 in RAW264.7 cells, indicating a pro-inflammatory effect of circEpc1 in RT-induced inflammatory injury and its potential value as a biomarker to for the diagnosis of RT poisoning.

Although it is not clear how circRNAs regulate the progression of inflammation, studies have shown that they may function as miRNA sponges to regulate the expression of target genes via the miRNA response element (J. Chen et al., 2019). It has been reported that CircBbs9 can promote PM_2.5_-induced lung inflammation by activating NLRP3 via sponge miR-30e-5p (M. Li et al., 2020). In addition, circGLIS2 abnormally activates NF-κB signaling pathway through miR-671 sponge mechanism in colitis cells (J. Chen et al., 2020). Moreover, circRNA, which is widely located in the cytoplasm, often acts as a miRNA sponge (Su et al., 2019). We confirmed that circEpc1 was mainly located in the cytoplasm by nucleocytoplasmic separation experiments. Furthermore, bioinformatics analysis showed that there were potential binding sites between circEpc1 and miR-5114. Our results showed that circEpc1 contains the binding site of miR-5114, and it could bind to miR-5114 in RT-treated RAW264.7 cells as verified by FISH and Dual-Luciferase reporter assays. In addition, we also constructed cell lines with miR-5114 overexpression using lentiviral vectors. Compared with the control cells, the secretion of TNF-α and IL-6 in miR-5114 overexpression cells was significantly downregulated. On the contrary, when co-transfected with miR-5114 overexpression and circEpc1 overexpression lentivirus vectors, this trend was reversed. Therefore, we concluded that miR-5114 is sponged by circEpc1 and plays a negative role in RT-induced inflammation.

To further explore the regulatory mechanism of circRNA as ceRNA, we identified a target gene NOD2 which can bind to miR-5114 through TargetScan. NOD2 belongs to the intracellular pattern-recognition receptors (PRRs) family, which can sense bacterial peptidoglycan (PGN) and muramyl dipeptide (MDP) (Girardin et al., 2003). Furthermore, NOD2 regulates multiple signaling pathways involved in a variety of cellular responses, including inflammatory responses via activation of NF-κB, MAPK, and type I IFNs, and autophagy (Trindade and Chen, 2020). Recent studies suggest that inhibition of NOD2 signaling pathway may be beneficial to the development of inflammatory disorders (Cavallari et al., 2017; Miller et al., 2018). Therefore, we thought that RT-induced RAW264.7 cells inflammation may due to NF-κB and MAPK activation downstream of NOD2 signaling pathway. Studies have shown that RIP2 is the essential adaptor kinase in NOD2 signaling pathway, and formation of the NOD2-RIP2 multiprotein complex after ligand recognition drives NF-κB/MAPK activation (Chin et al., 2002; Park et al., 2007). In this study, we found that the expressions of NOD2 and RIP2 were significantly upregulated in RT-treated RAW264.7 cells via qRT-PCR and WB analysis. Furthermore, the phosphorylation of IκB-α, p38, ERK, and JNK in RAW264.7 cells were increased following RT treatment. Studies on the regulatory mechanism of ceRNA confirmed that circEpc1 upregulated the expression of NOD2 and RIP2. Compared with the control cells, the phosphorylation of IκB-α, p38, ERK, and JNK in circEpc1 overexpression cells was significantly increased, while miRNA reversed these results. Above all, circEpc1 functions as a ceRNA to up-regulate the expression of NOD2 by competitive binding to miRNA-5114, leading to the progression of RT-induced inflammation via NF-κB and MAPK signaling pathways.

Presently, there are relatively few reports about the role of circRNAs in innate immunity and our study provided a novel insight into the role of circRNA in RT-induced inflammation. For this study, only the function of circEpc1 as a miRNA sponge has been explored. However, besides this mechanism, circRNAs can interact with RNA-binding protein to regulate gene expressions and encode functional proteins, indicating there may be other potential mechanisms for the role of circEpc1 in RT-induced inflammation and further research is needed.

## Conclusion

In summary, our findings reveals that circEpc1 competitively binds miR-5114 to abolish the suppressive effect of miR-5114 on NOD2, then promotes RT-induced inflammation via NF-κB and MAPK signaling pathways. The data provides a link between circRNAs, NOD2 signaling pathway and innate immunity. Based on this mechanism, it is believed that the circEpc1/miR-5114/NOD2 axis has great potential as a new biomarker and new therapeutic target for RT-induced inflammation in the future.

## Data Availability

The datasets presented in this study can be found in online repositories. The names of the repository/repositories and accession number(s) can be found below: https://www.ncbi.nlm.nih.gov/, GSE175619.

## References

[B1] Assiri A. S. (2012). Ricin Poisoning Causing Death after Ingestion of Herbal Medicine. Ann. Saudi Med. 32 (3), 315–317. 10.5144/0256-4947.2012.315 22588447 PMC6081027

[B2] Audi J. Belson M. Patel M. Schier J. Osterloh J. (2005). Ricin Poisoning: a Comprehensive Review. JAMA 294 (18), 2342–2351. 10.1001/jama.294.18.2342 16278363

[B3] Cavallari J. F. Fullerton M. D. Duggan B. M. Foley K. P. Denou E. Smith B. K. (2017). Muramyl Dipeptide-Based Postbiotics Mitigate Obesity-Induced Insulin Resistance via IRF4. Cell Metab 25 (5), 1063–e3. e1063. 10.1016/j.cmet.2017.03.021 28434881

[B4] Chen G. Shaw M. H. Kim Y. G. Nuñez G. (2009). NOD-like Receptors: Role in Innate Immunity and Inflammatory Disease. Annu. Rev. Pathol. 4, 365–398. 10.1146/annurev.pathol.4.110807.092239 18928408

[B5] Chen J. Liu G. Wu Y. Ma J. Wu H. Xie Z. (2019). CircMYO10 Promotes Osteosarcoma Progression by Regulating miR-370-3p/RUVBL1 axis to Enhance the Transcriptional Activity of β-catenin/LEF1 Complex via Effects on Chromatin Remodeling. Mol. Cancer 18 (1), 150. 10.1186/s12943-019-1076-1 31665067 PMC6819556

[B6] Chen J. Yang X. Liu R. Wen C. Wang H. Huang L. (2020). Circular RNA GLIS2 Promotes Colorectal Cancer Cell Motility via Activation of the NF-Κb Pathway. Cell Death Dis 11 (9), 788. 10.1038/s41419-020-02989-7 32968054 PMC7511409

[B7] Chin A. I. Dempsey P. W. Bruhn K. Miller J. F. Xu Y. Cheng G. (2002). Involvement of Receptor-Interacting Protein 2 in Innate and Adaptive Immune Responses. Nature 416 (6877), 190–194. 10.1038/416190a 11894097

[B8] Dong M. Yu H. Wang Y. Sun C. Chang Y. Yin Q. (2020). Critical Role of Toll-like Receptor 4 (TLR4) in Ricin Toxin-Induced Inflammatory Responses in Macrophages. Toxicol. Lett. 321, 54–60. 10.1016/j.toxlet.2019.12.021 31862508

[B9] Du W. W. Yang W. Liu E. Yang Z. Dhaliwal P. Yang B. B. (2016). Foxo3 Circular RNA Retards Cell Cycle Progression via Forming Ternary Complexes with P21 and CDK2. Nucleic Acids Res. 44 (6), 2846–2858. 10.1093/nar/gkw027 26861625 PMC4824104

[B10] Endo Y. Mitsui K. Motizuki M. Tsurugi K. (1987). The Mechanism of Action of Ricin and Related Toxic Lectins on Eukaryotic Ribosomes. The Site and the Characteristics of the Modification in 28 S Ribosomal RNA Caused by the Toxins. J. Biol. Chem. 262 (12), 5908–5912. 10.1016/s0021-9258(18)45660-8 3571242

[B11] Fridh V. Rittinger K. (2012). The Tandem CARDs of NOD2: Intramolecular Interactions and Recognition of RIP2. PLoS One 7 (3), e34375. 10.1371/journal.pone.0034375 22470564 PMC3314614

[B12] Girardin S. E. Travassos L. H. Hervé M. Blanot D. Boneca I. G. Philpott D. J. (2003). Peptidoglycan Molecular Requirements Allowing Detection by Nod1 and Nod2. J. Biol. Chem. 278 (43), 41702–41708. 10.1074/jbc.M307198200 12871942

[B13] Gresnigt M. S. Cunha C. Jaeger M. Gonçalves S. M. Malireddi R. K. S. Ammerdorffer A. (2018). Genetic Deficiency of NOD2 Confers Resistance to Invasive Aspergillosis. Nat. Commun. 9 (1), 2636. 10.1038/s41467-018-04912-3 29980664 PMC6035256

[B14] Guarnerio J. Bezzi M. Jeong J. C. Paffenholz S. V. Berry K. Naldini M. M. (2016a). Oncogenic Role of Fusion-circRNAs Derived from Cancer-Associated Chromosomal Translocations. Cell 166 (4), 1055–1056. 10.1016/j.cell.2016.07.035 27518567

[B15] Guarnerio J. Bezzi M. Jeong J. C. Paffenholz S. V. Berry K. Naldini M. M. (2016b). Oncogenic Role of Fusion-circRNAs Derived from Cancer-Associated Chromosomal Translocations. Cell 166 (2), 1055–1056. 10.1016/j.cell.2016.03.02010.1016/j.cell.2016.07.035 27518567

[B16] Han D. Li J. Wang H. Su X. Hou J. Gu Y. (2017). Circular RNA circMTO1 Acts as the Sponge of microRNA-9 to Suppress Hepatocellular Carcinoma Progression. Hepatology 66 (4), 1151–1164. 10.1002/hep.29270 28520103

[B17] Hansen T. B. Jensen T. I. Clausen B. H. Bramsen J. B. Finsen B. Damgaard C. K. (2013). Natural RNA Circles Function as Efficient microRNA Sponges. Nature 495 (7441), 384–388. 10.1038/nature11993 23446346

[B18] Hasegawa M. Fujimoto Y. Lucas P. C. Nakano H. Fukase K. Núñez G. (2008). A Critical Role of RICK/RIP2 Polyubiquitination in Nod-Induced NF-kappaB Activation. EMBO J. 27 (2), 373–383. 10.1038/sj.emboj.7601962 18079694 PMC2234345

[B19] Holdt L. M. Stahringer A. Sass K. Pichler G. Kulak N. A. Wilfert W. (2016). Circular Non-coding RNA ANRIL Modulates Ribosomal RNA Maturation and Atherosclerosis in Humans. Nat. Commun. 7, 12429. 10.1038/ncomms12429 27539542 PMC4992165

[B20] Jeck W. R. Sharpless N. E. (2014). Detecting and Characterizing Circular RNAs. Nat. Biotechnol. 32 (5), 453–461. 10.1038/nbt.2890 24811520 PMC4121655

[B21] Legnini I. Di Timoteo G. Rossi F. Morlando M. Briganti F. Sthandier O. (2017). Circ-ZNF609 Is a Circular RNA that Can Be Translated and Functions in Myogenesis. Mol. Cel 66 (1), 22–e9. e29. 10.1016/j.molcel.2017.02.017 PMC5387670 28344082

[B22] Lei K. Bai H. Wei Z. Xie C. Wang J. Li J. (2018). The Mechanism and Function of Circular RNAs in Human Diseases. Exp. Cel Res 368 (2), 147–158. 10.1016/j.yexcr.2018.05.002 29730164

[B23] Li M. Hua Q. Shao Y. Zeng H. Liu Y. Diao Q. (2020a). Circular RNA circBbs9 Promotes PM2.5-induced Lung Inflammation in Mice via NLRP3 Inflammasome Activation. Environ. Int. 143, 105976. 10.1016/j.envint.2020.105976 32707273

[B24] Li X. Jia Y. Nan A. Zhang N. Zhou H. Chen L. (2020b). CircRNA104250 and lncRNAuc001.dgp.1 Promote the PM2.5-induced Inflammatory Response by Co-targeting miR-3607-5p in BEAS-2B Cells. Environ. Pollut. 258, 113749. 10.1016/j.envpol.2019.113749 31864925

[B25] Li Y. Yin Z. Fan J. Zhang S. Yang W. (2019). The Roles of Exosomal miRNAs and lncRNAs in Lung Diseases. Signal. Transduct Target. Ther. 4, 47. 10.1038/s41392-019-0080-7 31728212 PMC6851157

[B26] Li Y. Zheng Q. Bao C. Li S. Guo W. Zhao J. (2015). Circular RNA Is Enriched and Stable in Exosomes: a Promising Biomarker for Cancer Diagnosis. Cell Res 25 (8), 981–984. 10.1038/cr.2015.82 26138677 PMC4528056

[B27] Liao P. Li Y. Li H. Liu W. (2016). Organellar Proteome Analyses of Ricin Toxin-Treated HeLa Cells. Toxicol. Ind. Health 32 (7), 1166–1178. 10.1177/0748233714549066 25227225

[B28] Lukiw W. J. (2013). Circular RNA (circRNA) in Alzheimer's Disease (AD). Front. Genet. 4, 307. 10.3389/fgene.2013.00307 24427167 PMC3875874

[B29] Memczak S. Jens M. Elefsinioti A. Torti F. Krueger J. Rybak A. (2013). Circular RNAs Are a Large Class of Animal RNAs with Regulatory Potency. Nature 495 (7441), 333–338. 10.1038/nature11928 23446348

[B30] Miller M. H. Shehat M. G. Alcedo K. P. Spinel L. P. Soulakova J. Tigno-Aranjuez J. T. (2018). Frontline Science: RIP2 Promotes House Dust Mite-Induced Allergic Airway Inflammation. J. Leukoc. Biol. 104 (3), 447–459. 10.1002/JLB.4HI0118-017RR 30052281 PMC6113092

[B31] Park J. H. Kim Y. G. McDonald C. Kanneganti T. D. Hasegawa M. Body-Malapel M. (2007). RICK/RIP2 Mediates Innate Immune Responses Induced through Nod1 and Nod2 but Not TLRs. J. Immunol. 178 (4), 2380–2386. 10.4049/jimmunol.178.4.2380 17277144

[B32] Rybak-Wolf A. Stottmeister C. Glažar P. Jens M. Pino N. Giusti S. (2015). Circular RNAs in the Mammalian Brain Are Highly Abundant, Conserved, and Dynamically Expressed. Mol. Cel 58 (5), 870–885. 10.1016/j.molcel.2015.03.027 25921068

[B33] Salzman J. Chen R. E. Olsen M. N. Wang P. L. Brown P. O. (2013). Cell-type Specific Features of Circular RNA Expression. Plos Genet. 9 (9), e1003777. 10.1371/journal.pgen.1003777 24039610 PMC3764148

[B34] Sowa-Rogozińska N. Sominka H. Nowakowska-Gołacka J. Sandvig K. Słomińska-Wojewódzka M. (2019). Intracellular Transport and Cytotoxicity of the Protein Toxin Ricin. Toxins 11 (6), 350. 10.3390/toxins11060350 PMC6628406 31216687

[B35] Su H. Tao T. Yang Z. Kang X. Zhang X. Kang D. (2019). Circular RNA cTFRC Acts as the Sponge of MicroRNA-107 to Promote Bladder Carcinoma Progression. Mol. Cancer 18 (1), 27. 10.1186/s12943-019-0951-0 30782157 PMC6379985

[B36] Tao M. Scacheri P. C. Marinis J. M. Harhaj E. W. Matesic L. E. Abbott D. W. (2009). ITCH K63-Ubiquitinates the NOD2 Binding Protein, RIP2, to Influence Inflammatory Signaling Pathways. Curr. Biol. 19 (15), 1255–1263. 10.1016/j.cub.2009.06.038 19592251 PMC2741418

[B37] Trindade B. C. Chen G. Y. (2020). NOD1 and NOD2 in Inflammatory and Infectious Diseases. Immunol. Rev. 297 (1), 139–161. 10.1111/imr.12902 32677123 PMC8928416

[B38] Xu N. Yuan H. Liu W. Li S. Liu Y. Wan J. (2013). Activation of RAW264.7 Mouse Macrophage Cells *In Vitro* through Treatment with Recombinant Ricin Toxin-Binding Subunit B: Involvement of Protein Tyrosine, NF-Κb and JAK-STAT Kinase Signaling Pathways. Int. J. Mol. Med. 32 (3), 729–735. 10.3892/ijmm.2013.1426 23820591

[B39] Yang Y. Gao X. Zhang M. Yan S. Sun C. Xiao F. (2018). Novel Role of FBXW7 Circular RNA in Repressing Glioma Tumorigenesis. J. Natl. Cancer Inst. 110 (3). 10.1093/jnci/djx166 PMC6019044 28903484

[B40] Yu J. Xu Q. G. Wang Z. G. Yang Y. Zhang L. Ma J. Z. (2018). Circular RNA cSMARCA5 Inhibits Growth and Metastasis in Hepatocellular Carcinoma. J. Hepatol. 68 (6), 1214–1227. 10.1016/j.jhep.2018.01.012 29378234

