# Supplementary material for: CircEpc1 Promotes Ricin Toxin-Induced Inflammation via Activation of NF-κB and MAPK Signaling Pathways by Sponging miR-5114
Source: Front Pharmacol. 2021 Oct 22;12:767900. doi: 10.3389/fphar.2021.767900 (PMC8569240; doi:10.3389/fphar.2021.767900)
Supplement: Supplementary file 1 [file Table1.DOC]

Table S1. Primer sequences

| **Name** | **Sequences** |
| --- | --- |
| β-actin | F: GTACCACCATGTACCCAGGC  R: AACGCAGCTCAGTAACAGTCC |
| mmu_circ_0000842 | F: GAAAGTTACACAGGAGTATCAGTCG  R: TGGGCTTCTTTTCATATTTGC |
| mmu_circ_0013121 | F: CTTATCCAACATTCTGCAAAGCATA  R: CTTTACACTGGGCTGACAACC |
| mmu_circ_0010862 | F: CAGCCAGCACTGGACAGATG  R: GCCTGGACCTTCTACTTTCTCTTT |
| mmu_circ_0007640 | F: GGACTACCCTTTACTCAAACAGGTT  R: GGCTCCTCTTCTTTGTCTTTGA |
| mmu_circ_0011990 | F: TTTCCAGTCCATTAACAAGCGT  R: CATAGTTGATTCCAAGTCAAAGTTC |
| mmu_circ_0001645 | F: TTAGGACTGTGAACAGGACGGT  R: GTCTGTCATCATGGGGAGCA |
| mmu_circ_0000663 | F: TCTGGAGTTGTTCTCTTTTTGGA  R: TGAAGGGTAGGAAGCAGTCTGT |
| mmu_circ_0002813 | F: GAAGAAGCGAATCCTACAGCAG  R: TCCGTCTCGGTCTTCCTCAC |
| NOD2 | F: CCATATCTGTGACGAAGG  R: GTAGGTGATGCCATTGTT |
| RIP2 | F: TCCAGAGTAAGAGGGAAGC  R: TCCTTGTAGGTTTGGTGC |
| U6 | F: CAAGGATGACACGCAAATTCG |
| mmu-miR-5114 | F: ACTGGAGACGGAAGCTGCAAGA |
